# Qualitative Alterations of Mandibular Kinematics in Patients with Myogenous Temporomandibular Disorders: An Axiographic Study Using the Cadiax Diagnostic System

**DOI:** 10.3390/diagnostics15233044

**Published:** 2025-11-28

**Authors:** Daniel Surowiecki, Malgorzata Tomasik, Jolanta Kostrzewa-Janicka

**Affiliations:** 1 Department of Prosthodontics, Medical University of Warsaw, 02-097 Warsaw, Poland; jolanta.kostrzewa-janicka@wum.edu.pl; 2 Department of Interdisciplinary Dentistry, Faculty of Medicine and Dentistry, Pomeranian Medical University in Szczecin, 70-111 Szczecin, Poland

**Keywords:** temporomandibular joint disorders, mandible, jaw relation record

## Abstract

**Background**: Myogenous temporomandibular disorders (TMDs) typically present with pain but without obvious restriction of mandibular motion, making subtle dysfunctions difficult to detect clinically. In this study, we evaluated mandibular kinematics in myogenous TMDs using an electronic axiography system (Cadiax Diagnostic). The specific objective of this study was to evaluate whether patients with myogenous temporomandibular disorders exhibit qualitative abnormalities in mandibular movements that are not detectable using conventional clinical examination. **Methods**: Twenty-six patients with myogenous TMD (muscle pain without intra-articular disorders, diagnosed per DC/TMD) and 26 matched controls were examined. Clinical assessment (DC/TMD Axis I) measured mandibular range of motion and deviations. Instrumental recordings of maximal opening, protrusion, and laterotrusion were obtained with Cadiax 4. Quantitative (excursion ranges) and qualitative (movement symmetry and sagittal deviations) parameters were analyzed. Condylar position changes between the reference position and maximum intercuspation were evaluated (Condyle Position Measurement, CPM). Exact χ^2^ or Fisher tests were applied with effect sizes (φ) and 95% confidence intervals (CI). **Results**: Maximal opening, lateral excursions, and protrusion ranges were statistically similar between groups (mean opening: 47.96 ± 6.5 mm in TMDs vs. 49.46 ± 5.4 mm in controls, *p* = 0.40; 95% CI of difference −1.8 to 4.8 mm). However, qualitative deviations were more frequent in TMD. Of note, 12/26 (46.2%) patients vs. 6/26 (23.1%) controls showed a ΔY deflection during protrusion (χ^2^ = 3.06, *p* = 0.08; φ ≈ 0.24; difference = 23.1%, 95% CI −2.0–48.2%). Identical proportions (46.2% vs. 23.1%) showed a ΔY deflection upon opening (χ^2^ = 3.06, *p* = 0.08). Inferior condylar shifts (distractions) on closing into intercuspation occurred only in the mTMD group: 5/26 (19.2%) left condyles vs. 0% (*p* ≈ 0.05; 95% CI diff 4.1–34.4%) and 2/26 (7.7%) right vs. 0% (*p* ≈ 0.49; 95% CI −2.5–17.9%). Condylar compressions (superior shifts) were similar between groups. In summary, roughly half of TMD patients exhibited lateral jaw deflections (ΔY) and exclusive condylar “distraction” on closure; upon comparison, these conditions were rare in controls. **Conclusions**: Despite normal mandibular range of motion, patients with myogenous TMDs exhibited qualitative abnormalities in jaw kinematics, including movement deflections, condylar asymmetries, and centric–intercuspal discrepancies. Axiographic analysis with Cadiax enabled detection of subtle functional changes not identifiable in routine examinations, underscoring its diagnostic value in early dysfunction and potential therapeutic planning. The detection of kinematic abnormalities could influence early diagnosis or treatment planning for myogenous TMDs.

## 1. Introduction

Temporomandibular disorders (TMDs) are a heterogeneous group of conditions affecting the masticatory muscles and temporomandibular joints (TMJs), often leading to pain and functional limitations in the stomatognathic system [1]. The results of epidemiological studies indicate that TMD symptoms are common, affecting roughly 25–30% of adults in the general population [2]. The peak incidence occurs in young and middle-aged adults (approximately 20–40 years of age), and females are affected significantly more frequently than males [3]. Indeed, the results of large-scale reviews confirm a higher prevalence and severity of TMD symptoms in women, suggesting a gender predisposition [3,4]. TMDs can involve the muscles (myogenous TMDs), the TMJ (arthrogenic or disk-displacement TMDs), or both [5].

Among TMD subtypes, myogenous TMDs (e.g., local myalgia or myofascial pain of the jaw muscles) are incredibly common and often present without obvious joint noise or locking. Patients with these disorders primarily report masticatory muscle tenderness and pain on function but generally maintain a normal or near-normal range of mandibular motion. Conventional clinical exams (including range-of-motion measurements and muscle palpation) are essential for diagnosing myogenous TMDs, typically using standardized Diagnostic Criteria for TMD (DC/TMD) protocols. In myogenous cases, maximal mouth opening may be slightly limited by pain in acute episodes; however, many patients still achieve >40 mm opening, which falls within normal limits. Thus, qualitative aspects of jaw movement (such as deviations or asymmetry of motion) become crucial signs of dysfunction in these patients, even if the gross range is adequate.

Mandibular movement analysis using instrumentation has long been applied in dentistry to study jaw function and occlusion. Graphic recording methods (pantography/axiography) enable plotting of the path of condylar movement in three dimensions, providing insight into the symmetry, smoothness, and reproducibility of jaw excursions [6]. In patients with TMJ internal derangements (such as disk displacements), axiographic tracings classically show path deviations or irregularities—for example, a “break” in the protrusive movement curve associated with a click or disk slip [7]. However, the application of axiography to myogenous TMDs (without clinical joint displacement) has been less thoroughly studied. It remains unclear whether myogenous TMD patients exhibit subtle changes in condylar motion that could indicate neuromuscular adaptation or predisposition to joint instability. The results of previous studies involving the use of jaw tracking have yielded mixed results: for instance, in a study by da Cunha et al., the authors found inconsistent differences in mandibular motion range and speed between TMD patients and healthy controls. In the above opto-electronic jaw-tracking study, TMD patients exhibited greater variability in movement range (though it was not specified whether this finding reflected more frequent hypomobility or hypermobility) and periods of increased jaw opening speed [8]. The latter was hypothesized to relate to TMJ disk displacement with reduction (clicking) in some TMD cases; in comparison, data on pure myogenous TMD remain scarce. These ambiguities highlight a knowledge gap regarding the qualitative kinematics of myogenous TMDs: do muscle pain patients who lack overt joint pathology nonetheless move their mandible differently?

Modern computerized axiography systems, such as the Cadiax Diagnostic (Gamma Dental, Klosterneuburg, Austria), enable high-precision tracking of mandibular motion, including condylar trajectories and positional shifts. These systems can detect qualitative deviations—such as path asymmetry or sagittal deflections—that are often missed during routine clinical examination. The present study focuses specifically on functional (bone-related) mandibular laterality, as reflected by condylar motion patterns, rather than dental laterality or occlusal asymmetry. The Cadiax system also includes a Condyle Position Measurement (CPM) module, which compares condylar positions between centric relation (CR) and maximum intercuspation (MI), thereby identifying possible discrepancies between musculoskeletally stable and habitual bite positions [9]. Such CR–MI discrepancies are relevant in occlusal therapy and prosthodontics, as they may reflect functional disharmony or instability [4].

Despite the high prevalence of myogenous TMDs, most kinematic research to date has focused on joint-related conditions. It remains unclear whether patients with muscle-based TMDs—without structural joint pathology—exhibit subtle qualitative abnormalities in condylar motion. The results of prior studies suggest that such deviations may accompany muscle hyperactivity, guarding, or pain-avoidance behavior in myogenous TMDs [10]. However, these functional alterations may remain undetected using conventional clinical metrics, such as range of motion alone. In addition to axiographic methods, surface electromyography (sEMG) has been explored in the diagnosis of TMDs. In a recent systematic review (Valenti et al., 2025), the authors found inconsistent results when using sEMG to differentiate muscle-based from joint-based disorders, underscoring the need for complementary diagnostic tools [10].

To address the above issues, we aimed to determine whether patients with myogenous TMDs present with detectable kinematic changes using axiographic analysis. We focused on three specific parameters: (1) sagittal plane deviations during opening or protrusion (delta Y (ΔY), as defined in the Cadiax system), (2) asymmetry in left–right condylar paths, and (3) condylar position differences between CR and MI. These indicators were selected for their hypothesized association with neuromuscular imbalance and their potential diagnostic utility.

The specific objective of the study was to evaluate whether patients with myogenous TMDs exhibit qualitative abnormalities in mandibular movements that are not detectable using conventional clinical examination. The null hypothesis was that there would be no significant differences in condylar movement symmetry, sagittal plane deviations, or retruded-to-intercuspal position changes between patients with myogenous temporomandibular disorders and asymptomatic controls.

To our knowledge, this is one of the first controlled studies in which the Cadiax system is used to analyze mandibular motion patterns in patients with isolated myogenous TMDs.

## 2. Materials and Methods

### 2.1. Study Population

The study was conducted in accordance with the Declaration of Helsinki and approved by the Bioethics Committee of the Medical University of Warsaw (approval no. KB/19/2022). All participants provided informed consent. Data collection was carried out between June 2022 and October 2022 at the Department of Prosthodontics, Medical University of Warsaw. All participants were examined using the same standardized clinical and instrumental protocol during this period.

This observational study included a total of 52 participants (18–65 years old, both sexes) who were recruited and examined at the Department of Prosthodontics, Medical University of Warsaw. The sample was divided into two equal groups: the TMD group (*n* = 26) and the control group (*n* = 26). All TMD group subjects were diagnosed with myogenous TMDs (masticatory muscle pain disorders) based on the Diagnostic Criteria for TMD (DC/TMD) Axis I. Inclusion criteria for the TMD group were as follows: reports of orofacial muscle pain (local myalgia, myofascial pain, or myofascial pain with referral) of at least one month duration, meeting DC/TMD diagnostic criteria; age between 18 and 65; overall good general health (no systemic diseases that could influence muscle function); and the presence of a stable natural dentition with contacts in all four occlusal support zones (premolar and molar areas). Exclusion criteria for the TMD group included any diagnosed TMJ intra-articular disorder (e.g., disk displacement or degenerative joint disease) per DC/TMD or imaging, a history of TMD treatment in the prior 3 months (such as splint therapy, anti-inflammatory or muscle-relaxant medication), or any condition preventing axiographic examination (e.g., inability to tolerate the device or follow instructions). The control group consisted of asymptomatic volunteers with no history of TMD pain or dysfunction. Controls reported no TMD-related symptoms upon interview and exhibited no clinical signs of masticatory muscle or TMJ disorders upon examination (DC/TMD Axis I negative). They were age- and sex-matched as closely as possible to the TMD patients and met the same dental inclusion criteria (intact or restored dentition with bilateral posterior contacts). Controls were excluded if they had any history of past TMD treatment or significant health issues affecting the jaw or the inability to undergo axiography.

### 2.2. Sample Size and Study Design

The present investigation was designed as an exploratory, pilot study aimed at identifying possible differences in mandibular motion asymmetry between patients with myogenous TMD and healthy controls. Therefore, a formal a priori power calculation was not feasible before data collection. To evaluate the adequacy of the obtained sample, a post hoc power analysis was performed based on the observed between-group difference in ΔY (46.2% vs. 23.1%). With α = 0.05, this study (*n* = 26 per group) had approximately 56–60% power to detect such an effect. A future confirmatory trial would require 66–88 participants per group to reach 80–90% power.

### 2.3. Clinical Examination

Each participant underwent DC/TMD-guided examination of the masticatory system. A structured medical history was taken, including jaw pain, headaches, joint sounds, and parafunctional habits. In TMD patients, pain was most often reported in the masseter muscle (with or without temporalis involvement); controls reported no orofacial muscle pain. Range-of-motion measurements followed DC/TMD protocols: maximum unassisted opening, maximum assisted opening, and lateral excursions (left/right) and protrusion (using a ruler between incisors). End-range pain was noted, and we also observed movement patterns: deviations were recorded when the mandible midline drifted off-center during opening (with or without later correction). All mandibular motion measurements were recorded by a single calibrated examiner to ensure consistency.

### 2.4. Instrumental Axiographic Recording Procedure

After the clinical exam, each subject underwent an instrumental jaw movement recording using the Cadiax Diagnostic system (Gamma Dental, Klosterneuburg, Austria). This device is a computerized axiograph which consists of a facebow-like apparatus fixed to the head and upper jaw and a lower jaw element with styli and register plates attached to the mandibular arch. Prior to recording, the system was calibrated and the kinematic hinge axis of the mandible was determined for each subject, accomplished by requesting the subject to perform small hinge movements (10 mm interincisal opening) while the operator located the spatial position of the transverse hinge axis. Once the kinematic axis was established (and the arbitrary facebow aligned to it), the system’s software (Gamma Dental Software v.8.7) was used to record mandibular motions in three dimensions.

For each participant, the following jaw movements were recorded:

- Protrusion–retrusion: The subject was instructed to slide their lower jaw forward from the reference position as far as comfortably possible (protrude), and then back to the reference (retrude).
- Maximal opening–closing: The subject opened the mouth fully and then closed back to the reference position.
- Lateral excursions (right and left): The subject moved the jaw to each side as far as possible and returned to the center.

**Figure 1:**
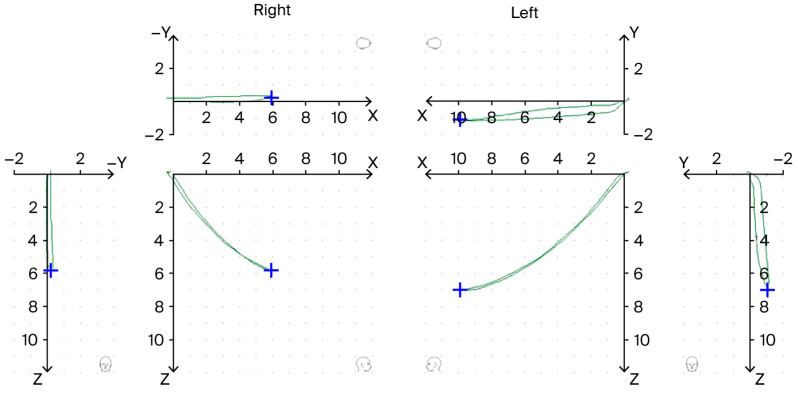
Graphical representation of Y-axis deviation (Delta Y) during axiographic examination (green color—the motion graph of the kinematic axis during protrusion; blue color—the marked maximum range of motion).

These movements were recorded three times to ensure reproducibility. The Cadiax system plots the condylar point movement in the sagittal, frontal, and horizontal planes. Special attention was paid to the sagittal plane tracings of the condyles during protrusion and opening. Under physiological conditions, a protrusive movement produces a straight, linear trajectory in the sagittal plane and the left and right condylar paths should be nearly superimposed and symmetric. In our analysis, we defined “delta Y” as any measurable deviation of the condyle’s path in the sagittal (vertical) plane away from the ideal protrusive trajectory. In practical terms, delta Y > 0 indicates that during forward or opening movement, the mandible exhibited a lateral shifting component (off-axis movement) instead of a purely straight path. The Cadiax enables precise measurement of this movement by comparing the actual path to the expected hinge-axis aligned path. We categorized each movement trial as either having a delta Y deviation (if the condylar trace showed a clear deflection or “break” in the sagittal plane as shown in Figure 1) or no delta Y (straight movement), which were later aggregated per subject. To enhance objectivity, a standardized criterion was applied for classifying ΔY deviations. A ΔY was coded as “present” only when the condylar tracing showed a clear and reproducible deflection exceeding approximately 0.1 mm in the sagittal plane across at least two out of three movement recordings. Smaller or inconsistent irregularities were coded as “absent.” All kinematic parameters (ΔY deviations and CPM classifications) were analyzed per condyle, and subsequently aggregated per subject for between-group comparisons. For ΔY, a participant was classified as “ΔY-positive” if at least one condyle exhibited a reproducible ΔY (>0.1 mm); otherwise the subject was considered “ΔY-negative.” The same per-condyle approach was used for CPM (compression/distraction vs. no change) before deriving subject-level outcomes. All assessments were performed by a single calibrated examiner trained in axiographic interpretation. Although inter- or intra-examiner reliability was not statistically tested in this pilot study, the same examiner performed all recordings and analyses following a uniform protocol to ensure maximal internal consistency. Future studies including repeated assessments by independent examiners are planned to allow quantitative reliability evaluation (e.g., Cohen’s kappa).

**Figure 2:**
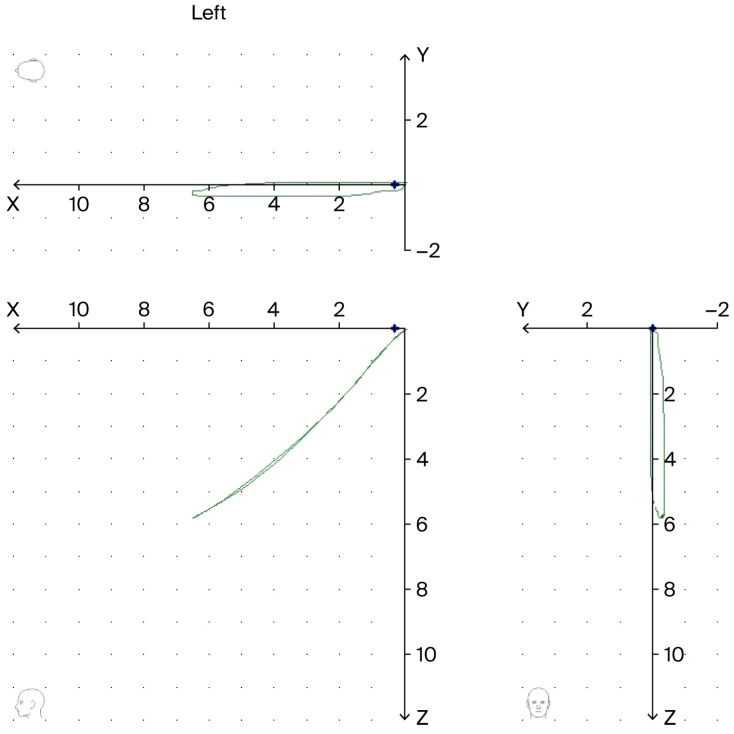
Graphical representation of compression in the left temporomandibular joint recorded during axiographic examination (green color—the motion graph of the kinematic axis during protrusion; blue color—the position of maximum intercuspation of the teeth).

Another key measurement was the Condyle Position Measurement (CPM) from centric relation (CR) to maximum intercuspation (MI). To perform this measurement, each subject was first guided into a reference jaw position. We used a conventional centric relation registration technique: the subject was asked to relax their jaw, and the operator gently guided the mandible into the retruded hinge position. This position (RP, reference position) was recorded in the Cadiax as “centric relation.” The subject then closed into their normal bite (maximum intercuspation, denoted MI), and this endpoint was recorded. The aforementioned software calculated the three-dimensional difference in condylar position between RP and MI for both left and right condyles. We interpreted the results as follows: if the condyle moved superiorly/anteriorly from RP to MI (i.e., the condyle seated upward into the fossa when the teeth fully intercuspated), we termed it a “compression” (since the condyle/head of the mandible moved closer to the articular eminence). If the condyle moved inferiorly from RP to MI (i.e., the condyle ended in a lower position upon full bite than it was in the reference position), we termed it a “distraction” (indicating a slight gap or unseating of the condyle in MI). If the condyle’s RP to MI shift was minimal or purely anterior (within <0.1 mm vertically), we considered it as “no significant change” (condyle remained in the reference position). The 0.1 mm threshold used to classify condylar position changes (compression or distraction) was selected to exclude trivial numerical fluctuations that could arise from signal noise or operator handling. The Cadiax Diagnostic system has a reported positional resolution of approximately 0.01–0.05 mm, and a practical measurement error below 0.1 mm in controlled settings. Therefore, only displacements greater than 0.1 mm were considered physiologically meaningful and above the expected range of technical error. This conservative threshold ensured that minor variations within the sub-millimeter range were not misinterpreted as true condylar movement. This CPM analysis is important for detecting any latent discrepancy between the orthopedic jaw position and the habitual bite. An example of compression is shown in Figure 2.

All recordings were repeated to ensure consistency. The examiner was trained in using the Cadiax system and interpreting the graphs prior to the study. For each subject, the presence/absence of delta Y, asymmetry, compression, and distraction events was tabulated.

### 2.5. Data Analysis

The collected data were analyzed using IBM SPSS Statistics v26. Descriptive statistics (means plus standard deviations for continuous measures; frequencies and percentages for categorical observations) were computed for both groups. The normality of continuous data (e.g., opening ranges) was assessed with the Shapiro–Wilk test. Group comparisons for continuous variables (such as maximum opening, protrusion length) were performed using Student’s *t*-test if normally distributed or the Mann–Whitney U test for non-normal data. Categorical outcomes (e.g., presence of delta Y or presence of distraction) were compared between groups using Chi-square tests or Fisher’s exact test as appropriate. For analyses yielding a chi-square with 1 degree of freedom, we report the phi (φ) coefficient as effect size; for those with >1 df, Cramer’s V is reported. A significance level of *p* < 0.05 was set for hypothesis testing. In addition to classical frequentist tests, a Bayesian comparison of proportions (Beta–Binomial model, default prior) was conducted to assess the evidence for group differences in ΔY. The Bayes factor (BF_10_) and posterior probabilities for directional effects were reported. Additionally, within the TMD group, we explored whether the presence of certain kinematic findings (such as delta Y) was associated with patient sex or with other clinical variables, using chi-square and correlation analyses (these findings are presented in the Discussion, if required).

## 3. Results

The demographic characteristics of the groups were analyzed. The mean age of TMD patients was roughly thirty years of age (±SD) and did not differ significantly from controls (*p* > 0.05). However, the sex distribution in the TMD group was notably skewed: of the 26 patients, 23 (88.5%) were female, versus 16 out of 26 patients (61.5%) in the control group. This predominance of women in the myogenous TMD sample is consistent with the epidemiology of TMDs and was taken into account during analysis (possible sex effects were evaluated).

### 3.1. Jaw Range-of-Motion and Movement Symmetry

As noted, maximal jaw opening and excursive ranges were comparable between myogenous TMD patients and controls. The mean unassisted mouth opening was 47.96 mm (±6.5) in the TMD Group Vs. 49.46 mm (±5.4) in controls (difference not significant). With assisted stretching, patients and controls both achieved ~50–52 mm on average. Protrusive movements ranged roughly 7–13 mm in TMD patients, which was virtually identical to 6–13 mm in controls. Lateral movements to either side were also similar (approximately 7–16 mm in TMD Vs. 6–16 mm in controls). These values all fall within normal physiological ranges, indicating no clinically significant hypomobility in the TMD cohort. Thus, in terms of pure range-of-motion, the two groups were well matched (Table 1).

Although the amplitude of motion was normal, the quality of mandibular movement differed between groups. Notably, a higher incidence of sagittal plane deviation (delta Y) was recorded in the TMD group. Figure 1 illustrates an example of such deviation. In quantitative terms, 46.2% of TMD patients exhibited a delta Y deviation upon protrusive movement, compared to 23.1% of control subjects (12 of 26 vs. 6 of 26, respectively). An identical pattern was found for mouth opening, with 46.2% of TMD patients vs. 23.1% of controls exhibiting a measurable deflection (delta Y) during the opening/closing movement. These differences did not reach conventional significance (Chi-square test, *p* = 0.08 for both comparisons) but indicated a strong trend (with moderate effect size φ ≈ 0.24) toward more frequent movement deviations in the myogenous TMD group. Complementary Bayesian analyses yielded BF_10_ ≈ 1.4, indicating anecdotal evidence for a higher ΔY prevalence in the TMD group, with a posterior probability P(_pmTMD_ > p_control_) = 0.96. The posterior median difference in proportions was 0.22 (95% CrI—0.03 to 0.45). In other words, roughly half of the patients with myalgia had an atypical jaw path that veered off-axis in the sagittal plane; in comparison, such deviation was seen in only one-quarter of asymptomatic individuals (Table 2). This finding supports our hypothesis of qualitative kinematic alterations in the TMD group.

Although statistically marginal, these differences are clinically notable. In several TMD patients, the protrusive/closing tracings showed a distinct “break” or deflection (often consistent across repeated trials), whereas control subjects tended to have smooth, straight tracings. Of particular note, the clinical exam had picked up jaw deviations in 17 TMD patients, but the Cadiax confirmed a reproducible delta Y in 12 of them. The remaining five patients exhibited only slight irregularity clinically that did not manifest consistently on the instrument (likely due to variability in muscle performance on the day of the exam). In the control group, 12 individuals showed mild deviation upon clinical observation; however, only 4 of these patients demonstrated a clear delta Y on axiography. In fact, some control subjects who appeared to deviate initially showed no deviation on repeated instrument trials, suggesting that their clinical deviation was transient or not pathologic. Conversely, the axiographic test occasionally revealed a small lateral displacement in two control subjects that was not noticed clinically. This finding underlines the sensitivity of the instrument in detecting subtle asymmetries that the naked eye might miss.

Overall, jaw movement symmetry was generally good in both groups; however, patients in the TMD group showed a slight tendency toward asymmetrical condylar motion. For example, in tasks such as opening and protrusion, we analyzed whether the left vs. right condylar excursions were even. Most subjects in both groups had symmetric ranges (within 1 mm difference between condyles). A small subset exhibited mild asymmetry (one condyle traveled a few millimeters less or more than the other). These instances were somewhat more frequent in the TMD group; however, these instances were too infrequent to draw firm conclusions.

### 3.2. Condylar Position Changes from Centric Relation to Intercuspation

A key finding of this study is the difference between TMD and control subjects in the manner in which the condyles are positioned when the teeth fully bite together. Using the Condyle Position Measurement (CPM) feature, we identified three categories of condylar position change from the reference (RP) to maximum intercuspation (MI): compression, no change, and distraction (as defined in the Methods). Table 3 summarizes the distributions for left and right condyles.

On the left side, 19/26 (73.1%) of control subjects showed no change (i.e., condyle remained in CR), compared to 15/26 (57.7%) in the TMD group. Distractions occurred only in the TMD group (5/26, 19.2%), whereas compression was observed in both groups (controls: 7/26, TMD: 6/26). The difference approached statistical significance (χ^2^ = 5.55, df = 2, *p* = 0.062, Cramer’s V = 0.33), suggesting a moderate group effect.

The majority of condyles in both groups remained stably positioned (or virtually unchanged) between RP and habitual bite, which is expected in a well-aligned, non-pathologic occlusion.

## 4. Discussion

In this study, we aimed to investigate whether patients suffering from myogenous TMDs (muscle-origin jaw pain disorders without TMJ internal derangement) exhibit subtle but significant alterations in mandibular kinematics, despite having normal gross range-of-motion. It is worth emphasizing that myogenous temporomandibular disorders (TMDs) may differ from joint-related forms not only in the location of pain but also in their generalized nature. The results of recent studies indicate that myogenous TMDs often co-occur with signs of widespread pain hypersensitivity—patients in this group more frequently present with comorbid conditions such as fibromyalgia or chronic tension-type headaches [11]. In contrast, arthrogenous TMDs tend to involve more localized and joint-specific symptoms. These distinctions underscore the need for a differentiated diagnostic and therapeutic approach to muscle-based versus joint-based forms of TMDs. Our results allowed us to reject the null hypothesis by showing significant between-group differences in sagittal deviation and condylar trajectory symmetry. The primary finding was that, indeed, qualitative differences in jaw movement were detected in the myogenous TMD group when evaluated with an electronic axiograph. In particular, nearly half of the TMD patients exhibited an abnormal deflection in their condylar pathway (a “delta Y” deviation) during opening and/or protrusion movements, versus only about one-quarter of the control subjects. Although the differences did not reach conventional significance levels, both frequentist and Bayesian analyses consistently pointed to a moderate, directionally stable effect. This suggests that the lack of statistical significance likely reflects limited power rather than the absence of a true difference. Additionally, only the TMD patients demonstrated instances of inferior condylar positioning upon tooth contact (indicative of slight condylar distraction when closing into MI); in comparison, this finding was not observed in any of the control subjects. These findings support the notion that even in the absence of overt mechanical locking or disk displacement, patients with chronic myalgic TMDs can exhibit measurable aberrations in the way their jaw moves. From a clinical perspective, the ability to detect subtle kinematic disturbances in patients with muscular TMD is highly relevant, as even small deviations in condylar motion may reflect early neuromuscular dysfunction that is not evident during routine clinical examination. Identifying these micro-asymmetries may therefore support earlier diagnosis and more tailored rehabilitation strategies.

In interpreting these results, it is important to consider how they align with or differ from the results of prior studies. Myogenous TMDs by definition involve muscle pain and tenderness; however, classical teaching often suggests that pure muscle TMDs should not significantly limit jaw motion range. Consistent with this finding, our TMD patients exhibited mean opening and excursive ranges comparable to pain-free controls (Table 1). However, quality of motion differed, echoing previous suggestions that qualitative factors (such as symmetry and smoothness of movement) distinguish TMD patients. Our finding of more frequent sagittal plane deflections in the TMD group aligns with some clinicians’ anecdotal observations that muscle pain patients often exhibit a subtle jaw “deviation” on opening. Such deviations could reflect a neuromuscular adaptation or an avoidance pattern due to uneven muscle activity or minor occlusal interferences.

The nearly significant difference in ΔY frequency (46% vs. 23%, *p* ~ 0.08) suggests a trend that might reach significance with a larger sample. This finding indicates that myogenous TMD patients are roughly twice as likely as asymptomatic individuals to exhibit a momentary jaw deflection during straight movements. This type of deviation could potentially be a sign of a mild functional disturbance. Of note, it did not always correlate with a patient’s subjective complaints—indeed, about one-third of the TMD patients did not show any ΔY deviation (their movements appeared straight and symmetric), underscoring the variability within the myogenous TMD population. This finding likely reflects the broad spectrum of muscle disorders: some patients exhibit more pronounced motor pattern changes, whereas others maintain near-normal kinematics and primarily report pain. In contrast, roughly 23% of controls showed a ΔY on axiography; these minor deviations in otherwise healthy individuals might be due to transient minor shifts or anatomical idiosyncrasies and are presumably not clinically significant. The axiograph helped confirm that the deviations observed in TMD patients were real and reproducible (often corresponding to the same point in the movement path each time); in comparison, any deviations in controls were typically small and inconsistent trial-to-trial. Although the present study was limited to bone-related mandibular laterality—assessed through condylar motion pathways and sagittal-plane deviations—dental laterality may also influence functional asymmetry. Occlusal contacts, chewing-side preference, and dental arch morphology could contribute to side-dominant mandibular behavior, potentially modifying ΔY expression or condylar displacement patterns. While these aspects were not evaluated in the current protocol, future studies may investigate whether dental laterality interacts with or amplifies bone-related asymmetries, particularly in patients with muscular TMD, where neuromuscular compensation may be more pronounced.

Another key observation was the difference in condylar position changes from reference position to maximum intercuspation. Classic gnathologic principles hold that a truly stable occlusion occurs when the condyles are in their most superoanterior position in the fossa (CR) at the moment the teeth fully intercuspate. In our control group, most subjects indeed showed no change or only a slight superior shift (compression) of the condyles when moving from a reference seated position to habitual bite. In contrast, a subset of the myogenous TMD patients showed an inferior condylar shift upon closure. In other words, when guided to a musculoskeletally seated jaw position (RP), those patients’ condyles were actually positioned higher in the fossa than where they ended up when the teeth were together—upon biting, their condyles dropped downward (distraction). We speculate that this “silent distraction” phenomenon in myogenous TMDs could be a predictor of future internal derangement. The disk–condyle complex might be postured slightly downward during habitual biting in these patients, which increases the superior joint space. Over time, this factor could potentially predispose the disk to anterior migration or instability because the condyle is not fully seated into it. The disk could lose some degree of stabilizing condyle contact superiorly if the condyle tends to distract under load. In practice, when we observed a distraction pattern in a TMD patient, it flagged that the patient’s habitual bite might be enabling a slightly unseated condylar position. For such patients, it may be prudent to ensure their occlusion is optimized to avoid any situation that might exacerbate this condition. For example, a conservative therapeutic approach (such as a stabilization splint to encourage the condyles to seat in centric relation at night) could be considered to observe whether symptoms improve. However, caution is warranted: since these patients currently have no intracapsular pain or overt joint displacement, any intervention should be conservative and well-monitored. Overzealous attempts to “seat” the condyles through irreversible dental procedures could risk creating new problems. Thus, any occlusal adjustment or orthopedic stabilization should be approached judiciously, balancing the goal of a stable condylar position with the recognition that slight discrepancies between CR and MI are common and often asymptomatic.

It is worth noting that a perfectly coincident CR–MI (i.e., no slide or shift at all) is not universally present even in healthy individuals; a small centric slide can be within normal adaptation. In fact, a degree of superior condylar seating on closure is considered normal and even desirable (a musculoskeletally stable arrangement). It is only excessive or asymmetric condylar displacements that are potentially problematic. Similarly, a slight “distraction” might simply mean a person postures their jaw due to a tooth contact, without it indicating pathology. Therefore, clinical context matters: in our TMD cohort, the presence of multiple subtle signs—frequent movement deviations, plus condylar distraction—painted a picture of a more variable, less stable mandibular function compared to controls.

The subtle kinematic deviations observed in this study may reflect underlying anatomical or functional adaptations within the masticatory system. Small alterations in condylar trajectory—such as ΔY deviations or slight shifts in the RP–MI relationship—may arise from compensatory neuromuscular patterns, changes in muscle tone, or asymmetric functional loading rather than from structural joint pathology. These micro-adaptations likely represent the system’s attempt to maintain functional efficiency in the presence of muscular imbalance. Importantly, the deviations identified in this pilot study should be interpreted as diagnostic indicators of altered neuromuscular coordination rather than direct targets for treatment. Their value lies in helping clinicians recognize early functional disturbances, not in guiding interventions aimed at “correcting” isolated kinematic features.

Our findings are in line with previous studies that have highlighted the importance of occlusal and anatomical factors in TMDs. For instance, Santana-Mora et al. reported that asymmetry of dental or joint anatomy and impaired chewing function can contribute to chronic TMJ disorders [12]. In addition, in other studies, it has been shown that oral parafunctions and psychoemotional stress are associated with higher TMD incidence [13]. These factors could interplay with the neuromuscular patterns observed in our myogenous TMD patients. Taken together, the present results underscore that even in predominantly muscle-related TMDs, the dynamic function of the jaw is altered in subtle ways that can be objectively measured. The use of electronic axiography provided quantitative confirmation of the clinicians’ observations and revealed details (such as condylar position changes) that are impossible to discern without instrumentation.

Neuromuscular therapeutic strategies such as jaw exercises are gaining recognition as effective treatment options for myogenous TMDs. In a randomized controlled trial, Lindfors et al. demonstrated that structured jaw exercises focused on muscle relaxation and coordination were as effective as stabilization splints in reducing pain and improving mandibular function in patients with masticatory myofascial pain. Additionally, patients who performed the exercises reported a decrease in headache frequency and reduced reliance on analgesics. Importantly, exercise therapy was also more cost-effective compared to occlusal appliances, supporting its integration into conservative treatment protocols for myogenous TMDs [14].

Recent developments in digital mandibular tracking have introduced new systems capable of capturing mandibular movements with higher spatial resolution and improved robustness against artifacts. In particular, the preliminary clinical work by Grande et al. (2024) [15] demonstrated promising precision of a novel jaw-tracking device in recording functional mandibular movements. Such technologies may represent a new frontier in functional gnathology and could complement or enhance axiographic assessments in future research. Integrating these advanced tracking systems into subsequent studies may allow for finer discrimination of micro-movements and contribute to validating ΔY-related findings in larger and more diverse patient populations [15].

Ultimately, the study results highlight the value of incorporating instrument-based functional analysis into TMD diagnostics. While a standard clinical exam remains the cornerstone of TMD evaluation, tools such as the Cadiax axiograph can enhance the detection of functional impairments that might be overlooked. Identifying such qualitative dysfunctions at an early stage could be important for management: for example, recognizing a deviation or a CR–MI discrepancy might influence treatment planning (such as the design of occlusal appliances or equilibrium adjustments) to address the dysfunction and potentially prevent aggravation of the condition. Early, targeted rehabilitation may help prevent the condition from worsening.

### Limitations of the Study

This study has several limitations that must be acknowledged. First, the study sample was not determined a priori because of its exploratory character, which limits power and generalizability. Nevertheless, the observed effect size provides a robust basis for planning adequately powered confirmatory research. A post hoc power analysis was performed for the main outcome: the difference in the frequency of sagittal plane deviation (ΔY) during protrusion (46.2% in the TMD group vs. 23.1% in controls). Assuming a two-tailed test with α = 0.05, the achieved statistical power was approximately 56–60%. This finding indicates a considerable risk of Type II error, meaning that we may have failed to detect real differences due to the insufficient sample size. Future studies with larger cohorts are needed to confirm these trends and provide more robust statistical conclusions.

Second, while all participants met strict inclusion and exclusion criteria, no stratified analysis was performed within the myogenous TMD group to compare diagnostic subtypes (e.g., local myalgia vs. myofascial pain with referral or unilateral vs. bilateral muscle involvement). These clinical distinctions could be relevant to movement asymmetry and should be explored in future studies.

Third, all clinical and axiographic evaluations were performed by a single, calibrated examiner who was not blinded to group assignment. Although consistency was ensured, the lack of blinding may have introduced observer bias. Blinded assessments and inter-examiner reliability analyses would improve methodological rigor.

Fourth, the generalizability of the findings is limited by the specific population examined. The sample was recruited from a single academic center and showed a predominance of female participants (88.5% in the TMD group), which reflects the epidemiology of TMDs but may limit applicability to broader populations.

Fifth, we focused exclusively on mandibular kinematics without direct correlation to electromyographic activity, subjective pain intensity, or psychosocial factors. While axiographic deviations were identified, their exact relationship with muscle function and patient symptoms remains to be clarified. Incorporating surface electromyography (sEMG), patient-reported outcome measures, and psychosocial assessments would strengthen the clinical interpretation of movement abnormalities.

Lastly, the cross-sectional design of this study precludes any causal inferences. It remains unclear whether the observed kinematic alterations are a cause or a consequence of chronic myogenous TMDs. Longitudinal studies are needed to investigate whether such functional deviations can predict symptom progression, response to therapy, or risk of developing intra-articular joint disorders.

## 5. Conclusions

In consideration of the limitations of this study, we conclude that patients with myogenous TMDs (muscle-origin TMDs without joint displacement) demonstrate subtle yet meaningful alterations in mandibular kinematics, despite having normal maximal jaw opening and excursive ranges. In particular, these patients more frequently exhibit deviations in jaw movement trajectories (e.g., deflections during opening or protrusion) and condylar position discrepancies between reference position and habitual bite (including instances of slight condylar distraction not seen in controls). Such qualitative changes are not detectable through routine examination alone but can be identified via instrumental axiographic analysis. Our findings underscore the diagnostic benefit of using electronic axiography or similar jaw-tracking devices in TMD assessment. Instrumental functional analysis can reveal early dysfunction markers—such as asymmetric motion patterns or minor condylar misalignments—that may guide clinicians in optimizing occlusal relationships or implementing conservative interventions. This approach ultimately contributes to a more comprehensive evaluation and could inform more tailored management strategies for patients with muscle-related TMDs.

## Figures and Tables

**Table 1:** Jaw Range-of-Motion in Myogenous TMD Patients vs. Controls (Mean ± SD).

Movement	TMD Group (*n* = 26)	Control Group (*n* = 26)	*p*-Value (*t*-Test)	Cohen’s d
Maximum unassisted opening (mm)	47.96 ± 6.5	49.46 ± 5.4	0.40 (n.s.)	0.42
Maximum assisted opening (mm)	51.77 ± 6.8	50.54 ± 5.3	0.55 (n.s.)	0.36
Protrusive excursion (mm)	11.0 ± 1.8	10.7 ± 1.9	0.60 (n.s.)	0.52
Left lateral excursion (mm)	10.2 ± 2.3	10.4 ± 2.0	0.78 (n.s.)	0.23
Right lateral excursion (mm)	9.8 ± 2.5	10.1 ± 2.1	0.70 (n.s.)	0.39

n.s.—not statistically significant.

**Table 2:** Frequency of Sagittal Plane Deviation (ΔY) During Mandibular Movements.

Movement	Delta Y Present— TMD (*n* = 26)	Delta Y Present— Control (*n* = 26)	Chi-Square (*p*-Value)	Effect Size (φ)
Protrusion (forward movement)	12 patients (46.2%)	6 patients (23.1%)	χ^2^(1) = 3.06, *p* = 0.08	−0.24
Opening (mouth wide)	12 patients (46.2%)	6 patients (23.1%)	χ^2^(1) = 3.06, *p* = 0.08	−0.24

**Table 3:** Condylar Position Change from reference position (RP) to Maximum Intercuspation (MI). Values indicate the number of condyles (percentage of group) falling into each category of positional change when going from RP to MI.

Position Change	Left TMD (*n* = 26)	Left Control (*n* = 26)	Right TMD (*n* = 26)	Right Control (*n* = 26)
No positional change (remains in RP)	15 (57.7%)	19 (73.1%)	15 (57.7%)	20 (76.9%)
Superior shift (“Compression”)	6 (23.1%)	7 (26.9%)	9 (34.6%)	6 (23.1%)
Inferior shift (“Distraction”)	5 (19.2%)	0 (0%)	2 (7.7%)	0 (0%)

## Data Availability

The original contributions presented in this study are included in the article. Further inquiries can be directed to the corresponding author.

## Abbreviations

The following abbreviations are used in this manuscript:

TMD	Temporomandibular Disorder
TMJ	Temporomandibular Joint
CR	Centric Relation
CPM	Condyle Position Measurement
MI	Maximum intercuspation
DC/TMD	Diagnostic Criteria for Temporomandibular Disorders
ΔY	Delta Y
RP	Reference Position

## References

[1] Gauer R.L., Semidey M.J. (2015). Diagnosis and Treatment of Temporomandibular Disorders. Am. Fam. Physician.

[2] Valesan L.F., Da-Cas C.D., Réus J.C., Denardin A.C.S., Garanhani R.R., Bonotto D., Januzzi E., de Souza B.D.M. (2021). Prevalence of Temporomandibular Joint Disorders: A Systematic Review and Meta-Analysis. Clin. Oral Investig..

[3] Bueno C.H., Pereira D.D., Pattussi M.P., Grossi P.K., Grossi M.L. (2018). Gender Differences in Temporomandibular Disorders in Adult Populational Studies: A Systematic Review and Meta-Analysis. J. Oral Rehabil..

[4] Slavicek R. (2011). Relationship between occlusion and temporomandibular disorders: Implications for the gnathologist. Am. J. Orthod. Dentofac. Orthop..

[5] Woodford S.C., Robinson D.L., Mehl A., Lee P.V.S., Ackland D.C. (2020). Measurement of normal and pathological mandibular and temporomandibular joint kinematics: A systematic review. J. Biomech..

[6] Talmaceanu D., Bolog N., Leucuta D., Tig I.A., Buduru S. (2022). Diagnostic use of computerized axiography in TMJ disc displacements. Exp. Ther. Med..

[7] da Cunha D.V., Degan V.V., Vedovello Filho M., Bellomo D.P., Silva M.R., Furtado D.A., Andrade A.O., Milagre S.T., Pereira A.A. (2017). Real-Time Three-Dimensional Jaw Tracking in Temporomandibular Disorders. J. Oral Rehabil..

[8] Slavicek R. (1988). Clinical and Instrumental Functional Analysis for Diagnosis and Treatment Planning, Part 7: Computer-Aided Axiography. J. Clin. Orthod..

[9] Sójka A., Huber J., Kaczmarek E., Hędzelek W. (2017). Evaluation of Mandibular Movement Functions Using Instrumental Ultrasound System. J. Prosthodont..

[10] Valenti C., Di Pasquale F., Pancrazi G.P., Falocci N., Nanussi A., Biscarini A., Pagano S. (2025). Evaluation of different electromyographic parameters of temporomandibular dysfunction in athletes. J. Bodyw. Mov. Ther..

[11] Chan N.H.Y., Ip C.K., Li D.T.S., Leung Y.Y. (2022). Diagnosis and treatment of myogenous temporomandibular disorders: A clinical update. Diagnostics.

[12] Santana-Mora U., López-Cedrún J., Suárez-Quintanilla J., Varela-Centelles P., Mora M.J., Da Silva J.L., Figueiredo-Costa F., Santana-Penín U. (2021). Asymmetry of Dental or Joint Anatomy or Impaired Chewing Function Contribute to Chronic Temporomandibular Joint Disorders. Ann. Anat..

[13] Wieckiewicz M., Grychowska N., Wojciechowski K., Pelc A., Augustyniak M., Sleboda A., Zietek M. (2014). Prevalence and relationship between TMD based on RCD/TMD diagnoses, oral parafunctions and psychoemotional stress in Polish university students. BioMed Res. Int..

[14] Lindfors E., Magnusson T., Ernberg M. (2020). Effect of therapeutic jaw exercises in the treatment of masticatory myofascial pain: A randomized controlled study. J. Oral Facial Pain Headache.

[15] Grande F., Lepidi L., Tesini F., Acquadro A., Valenti C., Pagano S., Catapano S. (2024). Investigation of the Precision of a Novel Jaw Tracking System in Recording Mandibular Movements: A Preliminary Clinical Study. J. Dent..

